# Supplementary material for: Unravelling the Shared Genetic Mechanisms Underlying 18 Autoimmune Diseases Using a Systems Approach
Source: Front Immunol. 2021 Aug 13;12:693142. doi: 10.3389/fimmu.2021.693142 (PMC8415031; doi:10.3389/fimmu.2021.693142)
Supplement: Supplementary file 1 [file DataSheet_1.docx]

Supplementary Material

# Supplementary Data

The data that support the findings of this study are available in figshare with unique identifiers.

Supplementary data 1 - DOI: <https://doi.org/10.17608/k6.auckland.14273606>

Supplementary data 2 - DOI: <https://doi.org/10.17608/k6.auckland.14273630>

Supplementary data 3 - DOI: <https://doi.org/10.17608/k6.auckland.14273633>

Supplementary data 4 - DOI: <https://doi.org/10.17608/k6.auckland.14273654>

Supplementary data 5 - DOI: <https://doi.org/10.17608/k6.auckland.14274659>

Supplementary data 6 - DOI: <https://doi.org/10.17608/k6.auckland.14274794>

Supplementary data 7 - DOI: <https://doi.org/10.17608/k6.auckland.14287652>

Supplementary data 8 - DOI: <https://doi.org/10.17608/k6.auckland.14288042>

Supplementary data 9 - DOI: <https://doi.org/10.17608/k6.auckland.14288300>

Supplementary data 10 - DOI: <https://doi.org/10.17608/k6.auckland.14288834>

Supplementary data 11 - DOI: <https://doi.org/10.17608/k6.auckland.14289158>

Supplementary data 12 - DOI: <https://doi.org/10.17608/k6.auckland.14287337>

**2 Supplementary Figures**

**Figure 1. AiD associated SNPs lie within non-coding regions of the genome. A.**The majority of SNPs (eQTLs) associated with a change in expression of genes are present in the intergenic (a stretch of non-coding DNA sequence between two consecutive genes) and intronic (within non-coding regions inside the genes) regions of the genome (Supplementary data 4 Table 1).
